# Multi-Source Weighted Localization Based on Cascaded DOA-TDOA

**DOI:** 10.3390/s26051614

**Published:** 2026-03-04

**Authors:** Jinshen Fang, Jianfeng Li, Shenghui Zhao, Biyuan Xu

**Affiliations:** College of Electronic and Engineering, Nanjing University of Aeronautics and Astronautics, Nanjing 210016, China; jinshenf@nuaa.edu.cn (J.F.); zhaoshenghui@nuaa.edu.cn (S.Z.); xbysx2404098@nuaa.edu.cn (B.X.)

**Keywords:** DOA, TDOA, multi-source localization, beam forming

## Abstract

Time Difference of Arrival (TDOA)-based localization is widely used for its stability and high accuracy. However, in multi-source scenarios, TDOA measurements from multiple sources become entangled, making it difficult to separate and correctly associate them for accurate localization. To address this challenge, this paper proposes a cascaded DOA-TDOA-based multi-source weighted localization algorithm that leverages the strengths of Direction of Arrival (DOA)-based methods for separating multi-source signals and the high precision of TDOA-based methods for single-source localization. The proposed method first estimates the DOAs of multiple sources and performs DOA matching based on geometric consistency to obtain initial coarse position estimates. Subsequently, it applies wideband spatial filtering to wideband signals using the Minimum Variance Distortionless Response (MVDR) to separate multi-source signals, enhance the signal-to-noise ratio (SNR), and thereby guide the selection of the reference station and the performance of TDOA estimation. Then, TDOA estimation is performed, while the weights are assigned based on the difference in GDOP (D-GDOP), computed from the initial coarse estimate, and a weighted least-squares (WLS) method is applied to obtain the refined estimate. Finally, the D-GDOP of the refined estimate can be computed and used to reassign weights, yielding more accurate position estimate. Simulation results validate the effectiveness of the proposed method, showing superior estimation accuracy and robustness relative to existing approaches.

## 1. Introduction

Source localization is a critical research topic with broad applications across diverse domains, including resource exploration, emergency response, navigation, crustal deformation monitoring, and real-time task scheduling [1]. In recent years, the rapid advancement of electronic and wireless communication technologies has led to a proliferation of unauthorized emitters and unknown interference sources, posing significant threats to both public safety and national security. Passive localization systems have emerged as a promising solution to this challenge. Owing to their long detection range, operational concealment, and strong anti-jamming capabilities, they have been extensively investigated and are widely deployed in electronic warfare and countermeasure applications, where high localization accuracy and robustness are paramount. Given these demanding requirements, achieving high-resolution passive source localization remains an urgent and challenging problem that warrants further research.

Conventional source localization methods typically follow a two-stage paradigm: first, measurement parameters (such as DOA, TDOA) are extracted from signals captured by distributed sensor arrays; subsequently, these parameters are fused within a geometric or statistical framework to perform localization [2]. Two-step localization techniques are commonly categorized according to the physical parameters they exploit, including time-based methods such as Time of Arrival (TOA) [3,4] and TDOA [5], DOA approaches [6,7], and signal-strength-based Received Signal Strength (RSS) schemes [8]. While TOA-based localization necessitates precise clock synchronization among all receiving nodes—a stringent requirement in many practical deployments—RSS-based methods rely heavily on accurate prior knowledge of both the channel propagation characteristics and the transmitted signal power, assumptions that are frequently violated in real-world scenarios.

In contrast, DOA-based source localization presents several practical advantages, notably its structural simplicity and low computational overhead. Recent advances have further enhanced the performance and applicability of this paradigm. For instance, spatial position estimation has been improved through Taylor-series expansions to iteratively refine location estimates [9], while bias compensation strategies incorporating known noise statistics in angular measurements have been shown to reduce estimation errors [10]. To mitigate hardware limitations and resolve phase ambiguities inherent in array processing, beamspace ESPRIT-based multi-source DOA algorithms have been developed, effectively circumventing constraints imposed by a limited number of radio frequency (RF) chains and unknown phase offsets [11]. Moreover, sparse Bayesian learning (SBL) frameworks have been employed to achieve robust vehicle positioning: such methods typically begin with an initial estimate of the noise covariance via least-squares, refine the angular search grid using polynomial rooting, and then iteratively cross-localize based on updated DOA estimates [12].

TDOA-based localization, meanwhile, is widely recognized for its high accuracy, rapid convergence, and resilience to measurement noise. A number of algorithmic innovations have sought to approximate maximum likelihood (ML) performance under realistic conditions. The Two-Step Weighted Least-Squares method, for example, provides a computationally efficient solution that closely approximates the ML estimator [13]. Building on this, the classical Chan algorithm offers a two-stage approach that first computes a coarse estimate and subsequently refines it through ML optimization [14]. Further improvements include convex relaxation techniques applied to the ML formulation to yield robust TDOA estimators [15], as well as semidefinite programming (SDP)-based methods that jointly localize multiple sources without requiring carefully chosen initial values [16]. Notably, recent work has also addressed non-line-of-sight (NLOS) errors by reformulating the TDOA model as an equivalent TOA problem and introducing novel SDP constraints to suppress NLOS-induced biases [17].

Given the inherent limitations of single-parameter approaches, multi-information fusion strategies that integrate complementary measurements—such as DOA and TDOA—have attracted considerable research interest. A representative two-stage hybrid passive localization framework first acquires both DOA and TDOA observations and then solves a system of nonlinear equations to estimate the emitter’s position [18]. This general strategy underpins most contemporary hybrid methods, which primarily focus on effective linearization or numerical solution techniques for the resulting nonlinear system. In one geometry-inspired approach, virtual sites are introduced to transform NLOS reflection paths into equivalent line-of-sight (LOS) trajectories, thereby enabling an initial coarse estimate; subsequent refinement is achieved by linearizing the joint TDOA/DOA observation equations to improve localization accuracy [19].

This paper proposes a cascaded DOA-TDOA-based multi-source weighted localization algorithm that leverages angular information as auxiliary data. By employing beam forming for spatial filtering, the method enhances the performance of time-difference estimation, thereby achieving more accurate TDOA-based localization. The main contributions are as follows:

- Unlike most existing localization systems that fuse DOA and TDOA information in a fusion function whose performance may be limited by the imbalance between errors in these measurements, this paper proposes a novel approach that uses DOA measurements in a new way to improve the accuracy of TDOA measurements. By fully exploiting the array’s beam-pattern, wideband spatial filtering is applied at the array output to suppress interference and significantly improve the SNR in TDOA localization.
- The proposed method effectively combines the high-precision advantage of TDOA-based single-source localization with the source-separation capability of DOA-based localization in multi-source scenarios. By utilizing DOA information to guide the selection of the reference station and TDOA estimation, and obtaining a coarse position estimate to compute D-GDOP, this approach addresses both the weight assignment problem in WLS and the TDOA data entanglement issue in multi-source environments.
- Comprehensive performance comparisons are presented between the proposed algorithm and conventional approaches—including K-means clustering, TDOA-only localization, and the TDOA-DOA joint least-squares (LS) method—across various parameter settings. Extensive simulations demonstrate the effectiveness and reliability of the proposed scheme.

## 2. Problem Formulation

**Figure 1:**
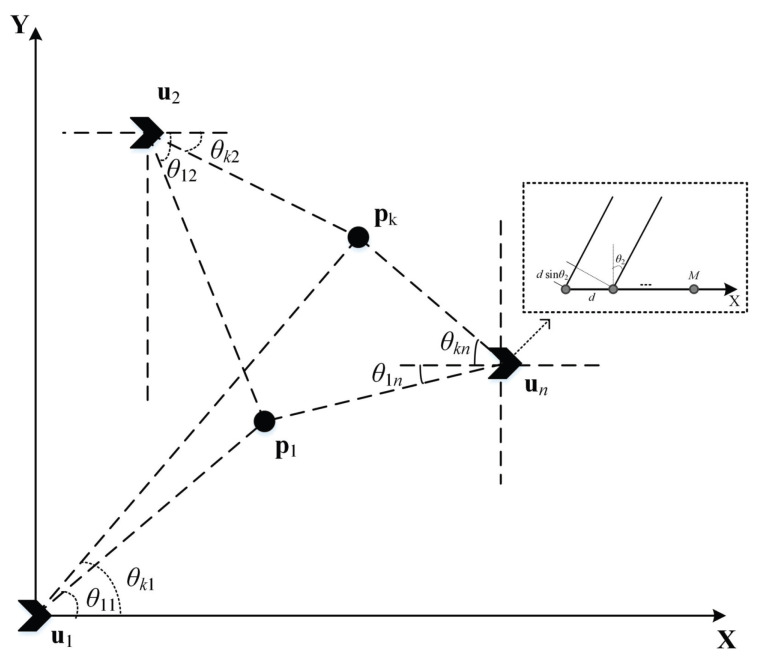
System model of multi-source Weighted Localization based on cascaded DOA-TDOA.

We consider the localization scenario depicted in Figure 1, where distributed arrays are employed in a two-dimensional plane. It is assumed that there are *N* stationary observation stations with precisely known positions in space, located at $u_{n}={\left[x_{n},y_{n}\right]}^{T},n=1,2,\ldots ,N$, each observation station is equipped with an ideal uniform linear array consisting of *M* elements. All elements are considered to be indistinguishable points, and mutual coupling between the elements is neglected. In space, there are *K* far-field radiation sources located at $p_{k}={\left[v_{k},w_{k}\right]}^{T}$. It is assumed that the number of radiation sources *K* is known.

Assume that the signal from each radiation source is a far-field wideband signal. The DOAs of the *k*-th radiation source at the *n*-th observation station are denoted as $\theta_{k,n}\in \left(-\pi /2,\pi /2\right)$. The output of the *m*-th element in the *n*-th array can be expressed as:

(1) $$x_{n,m}\left(t\right)=\sum_{k=1}^{K}s_{k}\left(t-\tau_{knm}\right)+b_{n,m}\left(t\right)m=1,2,\cdots ,M$$

where $s_{k}\left(t\right)$ denotes the *k*-th interfering source signal, $b_{n,m}\left(t\right)$ is the additive noise at the *m*-th element, and $\tau_{k,n,m}$ is the time delay of the *k*-th interfering source signal arriving at the *m*-th element of the *n*-th array relative to the first element, given by $\tau_{knm}=\left(m-1\right)d\sin\theta_{k,n}/c$ where *d* is the inter-element spacing of the uniform linear array (ULA), and *c* is the signal propagation speed in the medium.

For wideband signal processing, the most common approach is to uniformly partition the signal into multiple non-overlapping frequency sub-bands in the frequency domain. After such partitioning, the signal within each sub-band can be treated as narrowband. Therefore, by applying the Discrete Fourier Transform (DFT) to the output of each array element, the frequency-domain representation of the element outputs can be expressed as:

(2) $$X_{n,m}\left(\omega \right)=\sum_{k=1}^{K}S_{k}\left(\omega \right)e^{-j\omega \tau_{km}}+B_{n,m}\left(\omega \right)\;\;\;\;,\;\;\;\;\;\;\;\;m=1,2,\cdots ,M$$

where $X_{n,m}\left(\omega \right)$, $S_{k}\left(\omega \right)$, and $B_{n,m}\left(\omega \right)$ are the frequency-domain representations of $x_{n,m}\left(t\right)$, $s_{k}\left(t\right)$, and $b_{n,m}\left(t\right)$, respectively, obtained by applying the DFT.

By combining the received signals from all array elements, the array output model in the frequency domain for all received data can be expressed as:

(3) $$\left[\begin{array}{c} X_{n,1}\left(\omega \right)\\ X_{n,2}\left(\omega \right)\\ \vdots \\ X_{n,M}\left(\omega \right) \end{array}\right]=\left[\begin{array}{cccc} e^{-j\omega \tau_{1n1}} & e^{-j\omega \tau_{2n1}} & \cdots & e^{-j\omega \tau_{Kn1}}\\ e^{-j\omega \tau_{1n2}} & e^{-j\omega \tau_{2n2}} & \cdots & e^{-j\omega \tau_{Kn2}}\\ \vdots & \vdots & \ddots & \vdots \\ e^{-j\omega \tau_{1nM}} & e^{-j\omega \tau_{2nM}} & \cdots & e^{-j\omega \tau_{KnM}} \end{array}\right]\left[\begin{array}{c} S_{1}\left(\omega \right)\\ S_{2}\left(\omega \right)\\ \vdots \\ S_{K}\left(\omega \right) \end{array}\right]+\left[\begin{array}{c} B_{n,1}\left(\omega \right)\\ B_{n,2}\left(\omega \right)\\ \vdots \\ B_{n,M}\left(\omega \right) \end{array}\right]$$

The above equation can be rewritten as:

(4) $$X_{n}\left(\omega \right)=A_{n}\left(\omega ,\theta \right)S\left(\omega \right)+B_{n}\left(\omega \right)$$

To divide the wideband signal into multiple non-overlapping narrowband sub-bands in the frequency domain, an *J*-point DFT is applied to the received data in Equation (1). The resulting array reception model for the wid-eband signal can then be expressed as:

(5) $$X_{n}\left(\omega_{j}\right)=A_{n}\left(\omega_{j},\theta \right)S\left(\omega_{j}\right)+B_{n}\left(\omega_{j}\right),\;\;\;\;j=1,2,\cdots ,J$$

where $X_{n}\left(\omega_{j}\right)$ is the received signal data at angular frequency $\omega_{j}$, $S(\omega_{j})$ is the source signal data at angular frequency $\omega_{j}$, $B_{n}\left(\omega_{j}\right)$ is the noise data at angular frequency $\omega_{j}$, and $A_{n}\left(\omega_{j},\theta \right)$ is the array steering matrix at angular frequency $\omega_{j}$, which is given explicitly as follows:

(6) $$X_{n}\left(\omega_{j}\right)={\left[X_{n,1}\left(\omega_{j}\right),X_{n,2}\left(\omega_{j}\right),X_{n,3}\left(\omega_{j}\right),\cdots ,X_{n,M}\left(\omega_{j}\right)\right]}^{T}$$

(7) $$S\left(\omega_{j}\right)={\left[S_{1}\left(\omega_{j}\right),S_{2}\left(\omega_{j}\right),S_{3}\left(\omega_{j}\right),\cdots ,S_{K}\left(\omega_{j}\right)\right]}^{T}$$

(8) $$B_{n}\left(\omega_{j}\right)={\left[B_{n,1}\left(\omega_{j}\right),B_{n,2}\left(\omega_{j}\right),B_{n,3}\left(\omega_{j}\right)\cdots ,B_{n,M}\left(\omega_{j}\right)\right]}^{T}$$

(9) $$A_{n}\left(\omega_{j},\theta \right)=\left[a_{n,1}\left(\omega_{j},\theta \right),a_{n,2}\left(\omega_{j},\theta \right),a_{n,3}\left(\omega_{j},\theta \right),\cdots a_{n,K}\left(\omega_{j},\theta \right)\right]$$

(10) $$a_{n,k}\left(\omega_{j},\theta \right)={\left[e^{-j\omega_{j}\tau_{kn1}},e^{-j\omega_{j}\tau_{kn2}},e^{-j\omega_{j}\tau_{kn3}},\cdots ,e^{-j\omega_{j}\tau_{knM}}\right]}^{T}$$

The time-domain of received signal $X_{n}\left(t\right)$ is obtained by applying a *J*-point inverse DFT (IDFT) to $X_{n}\left(\omega \right)$.

## 3. The Proposed Method

In this section, we propose a cascaded DOA-TDOA-based multi-source weighted localization algorithm. The approach begins with DOA estimation, which is subsequently employed to steer a beamforming filter. This spatial filtering enhances the signal quality, yielding more accurate TDOA estimates and, consequently, improved localization accuracy.

### 3.1. DOA Estimation Based on DCS-SOMP

For narrowband signals, there are many commonly used DOA estimation methods, such as MUSIC, ESPRIT [20], etc., which will not be introduced here. For wideband signals, this paper presents a commonly used method called Distributed Compressive Sensing Simultaneous Orthogonal Matching Pursuit (DCS-SOMP) [21].

First, divide the bandwidth into *H* sub-bands, each with a center angular frequency of $\omega_{h}$ and an index set $G=\left\{\theta_{g}\right\},g=1,2,\cdots ,G$. Compute the array steering vector $A_{D}\left(h\right)$ corresponding to the index set of each sub-band as follows:

(11) $$A_{D}\left(h\right)=\left[a\left(\omega_{h},\theta_{1}\right),a\left(\omega_{h},\theta_{2}\right),\cdots ,a\left(\omega_{h},\theta_{G}\right)\right]$$

Initialize the residual $r_{0}=X_{n}$, the support set U=∅, and the iteration counter l=1.

Compute the joint correlation between each candidate atom index and the current residual, and select the atom with the maximum correlation as follows:

(12) $$g_{l}=\underset{g=1,2,\ldots ,G}{\arg\max}\sum_{h=1}^{H}\frac{\left|\langle r_{l-1},a\left(\omega_{h},\theta_{g}\right)\rangle \right|}{{∥a(\omega_{h},\theta_{g})∥}_{2}}$$

Update the support set:

(13) $$U=U\cup \left\{g_{l}\right\}$$

Update the residual:

(14) $${\hat{p}}_{h}={\left(A_{D}{\left(h,U\right)}^{T}A_{D}\left(h,U\right)\right)}^{-1}A_{D}{\left(h,U\right)}^{T}X_{n}$$

(15) $$r_{l}=X_{n}-A_{D}\left(h,U\right){\hat{p}}_{h}$$

where $A_{D}\left(h,U\right)$ denotes a set of columns of $A_{D}\left(h\right)$ collected by U.

If the number of iterations is less than *K*, continue iterating until l=K to obtain the angle estimation results:

(16) $${\hat{\alpha }}_{n}=\left\{\theta_{g}\right\},g\in U$$

### 3.2. Angle Matching Based on Geometric Consistency and Coarse Estimation

The DOA estimates ${\hat{\alpha }}_{n}$ obtained from the previous section can be expressed as $\left\{{\hat{\alpha }}_{1,n},{\hat{\alpha }}_{2,n},\cdots ,{\hat{\alpha }}_{K,n}\right\}$. However, since it is unknown which angle corresponds to which source, angle matching is required. In the following, geometric consistency is exploited to perform this angle matching.

The angle estimates from all arrays can be written in matrix form as

(17) $$\left.\left\{\begin{array}{c} {\hat{\alpha }}_{1,1},{\hat{\alpha }}_{1,2},\cdots ,{\hat{\alpha }}_{1,N}\\ \vdots \\ {\hat{\alpha }}_{K,1},{\hat{\alpha }}_{K,2},\cdots ,{\hat{\alpha }}_{K,N} \end{array}\right.\right\}$$

where each column represents the angle estimates from a single array corresponding to different radiation sources.

Next, one angle is selected from each column to form a combination. Using the known array positions and the angles in the selected combination, the coordinates of the intersection points are computed. Suppose there are *X* such combinations in total, and each combination yields *Y* intersection points. A cost function is then constructed as follows:

(18) $$L\left(x\right)=\sum_{i}^{Y-1}\sum_{j=i+1}^{Y}{∥p_{i}-p_{j}∥}_{2},x=1,2,\ldots ,X$$

where $p_{i}$ denotes the *i*-th intersection point.

The combination that minimizes the cost function is identified as the correctly matched set of angle estimates:

(19) $$x_{sort}=\underset{x}{\arg}\min\left(L\left(x\right)\right)$$

Based on the above procedure, the matched angle estimates are obtained, assumed to be:

(20) $$\left.\left\{\begin{array}{c} {\hat{\theta }}_{1,1},{\hat{\theta }}_{1,2},\cdots ,{\hat{\theta }}_{1,N}\\ \vdots \\ {\hat{\theta }}_{K,1},{\hat{\theta }}_{K,2},\cdots ,{\hat{\theta }}_{K,N} \end{array}\right.\right\}$$

The coarse estimate of the source can be obtained using the matched angles. Construct equations based on these DOAs:

(21) $$G_{ini,k}p_{ini,k}=h_{ini,k}$$

where

(22) $$G_{ini,k}=\left[\begin{array}{cc} -\tan({\hat{\theta }}_{k,1}) & 1\\ -\tan({\hat{\theta }}_{k,2}) & 1\\ \vdots & \vdots \\ -\tan({\hat{\theta }}_{k,N}) & 1 \end{array}\right],p_{ini,k}=\left[\begin{array}{c} x\\ y \end{array}\right],h_{ini,k}=\left[\begin{array}{c} y_{1}-x_{1}*\tan\left({\hat{\theta }}_{k,1}\right)\\ y_{2}-x_{2}*\tan\left({\hat{\theta }}_{k,2}\right)\\ \vdots \\ y_{N}-x_{N}*\tan\left({\hat{\theta }}_{k,N}\right) \end{array}\right]$$

Obtain the coarse source estimate using the least-squares method as

(23) $$p_{ini,k}={\left({G_{ini,k}}^{T}G_{ini,k}\right)}^{-1}{G_{ini,k}}^{T}h_{ini,k}$$

It is well known that the Generalized Cross-Correlation (GCC) method, when applied to TDOA estimation in multi-source scenarios, suffers from several limitations—most notably, inaccuracies in spectral peak localization. Even when accurate TDOA estimates for individual sources are obtained, correctly associating these estimates across sensor pairs and disambiguating them among multiple concurrent emitters remains a significant challenge. To address this issue, the proposed approach leverages matched DOA estimates to separate the sources, thereby enabling more reliable and source-specific TDOA estimation.

### 3.3. Beam Forming for Wideband Signal

The steering vector of a wideband signal depends not only on the DOA but also on the signal frequency. Consequently, conventional beamforming techniques—which assume a frequency-invariant steering vector—are applicable only to narrowband signals. Direct application of such methods to wideband signals results in distortion, including main lobe splitting and significant gain reduction. To mitigate these effects, wideband beamforming strategies must be employed.

First, the received array signals are transformed from the time domain to the frequency domain, yielding $X_{n}\left(\omega \right)$. Perform beam forming at each frequency bin:

(24) $${\hat{X}}_{k,n}\left(\omega \right)=w^{H}X_{n}\left(\omega \right)$$

where ${\hat{X}}_{k,n}$ represents the signal spectrum after beam forming. Since each beam forming operation processes only a single frequency bin, it is equivalent to performing beam forming on a narrowband signal. The weight vector w can be determined using the MVDR method [22] and expressed as:

(25) $$w=\frac{1}{a^{H}\left(\omega ,{\hat{\theta }}_{k,n}\right)R^{-1}a\left(\omega ,{\hat{\theta }}_{k,n}\right)}R^{-1}a\left(\omega ,{\hat{\theta }}_{k,n}\right)$$

where the matrix R is the covariance matrix of the array output.

### 3.4. Selection of Reference Station and TDOA Estimation Based on Generalized Cross-Correlation

As derived in the previous section, the set of beam-formed signal spectra at the output of each array is given by $\left\{{\hat{X}}_{k,1},{\hat{X}}_{k,2},\ldots ,{\hat{X}}_{k,N}\right\}$.

To mitigate degraded TDOA estimation accuracy caused by poor signal separability—arising, for instance, when multiple sources are proximate or exhibit collinearity with a particular observation station—it is essential to select an optimal reference station. Specifically, the reference station is chosen as the one that maximizes the angular separation among the sources relative to all other stations. This selection criterion enhances distinguishability, thereby promoting effective signal separation and improving the reliability of subsequent TDOA estimation.

**Figure 2:**
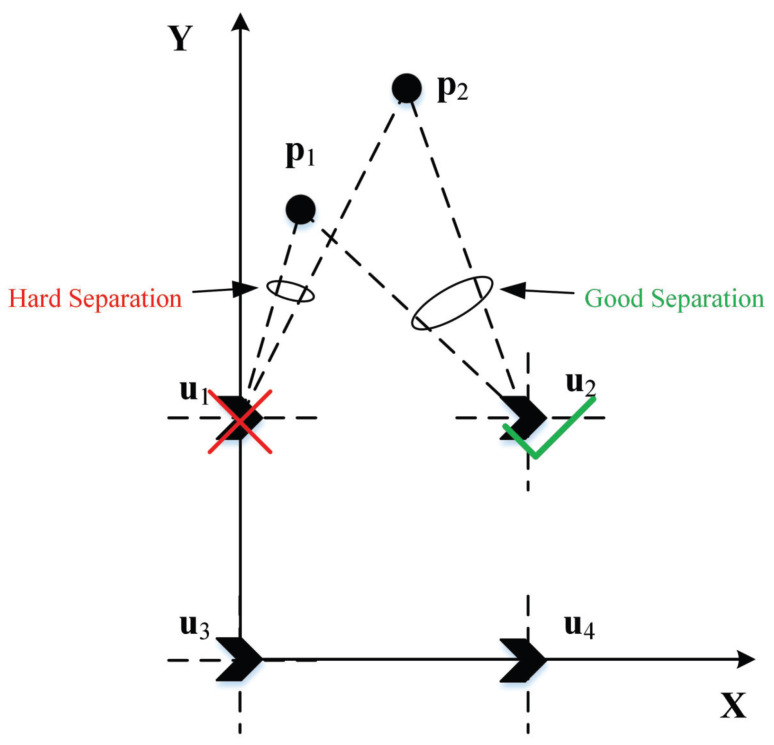
Reference station selection diagram.

As illustrated in Figure 2, the near collinearity of the two sources with $u_{1}$ hinders signal separation at that station, meaning $u_{1}$ would negatively impact TDOA estimation if used as the reference. In contrast, the significant angular separation at $u_{2}$ enables effective discrimination of the sources, making $u_{2}$ the preferred choice for the reference station.

Assume the output signal Spectrum of the first array ${\hat{X}}_{k,1}$ is taken as the reference. One signal from the remaining set is selected and cross-correlated with the reference signal using GCC for TDOA estimation. Denote the selected signal Spectrum as ${\hat{X}}_{k,n}$. According to the Wiener–Khinchin theorem, the cross-correlation function of two signals and their cross-power spectral density form a Fourier transform pair. Thus, the cross-correlation function between the output signal of array *n* and that of array 1 is given by

(26) $$R_{n,1}\left(\tau \right)=\frac{1}{\pi }\int_{0}^{\pi }G_{n,1}\left(\omega \right)e^{j\omega \tau }d\omega$$

where $G_{n,1}$ is the cross-power spectral density function of $x_{k,1}$ and $x_{k,n}$. To mitigate the effect of noise, a pre-filtering operation in the frequency domain is commonly applied. Here, we choose the PHAT weighting function as the pre-filtering function, expressed as

(27) $$\psi_{12}\left(\omega \right)=\frac{1}{{\hat{X}}_{k,1}\left(\omega \right)⊕{\hat{X}}_{k,n}^{*}\left(\omega \right)}$$

where ⊕ denotes the Hadamard product. The filtered cross-power spectral density function can then be expressed as

(28) $$G_{g1n}\left(\omega \right)=\psi_{12}\left(\omega \right)⊕G_{n,1}\left(\omega \right)$$

Again, according to the Wiener–Khinchin theorem, the filtered generalized cross-correlation function can be obtained as

(29) $$R_{g1n}\left(\tau \right)=\frac{1}{\pi }\int_{0}^{\tau }\psi_{12}\left(\omega \right)⊕G_{n,1}\left(\omega \right)e^{j\omega \tau }d\omega$$

After obtaining the generalized cross-correlation function, the time-difference estimation is given by the location of its peak, denoted as

(30) $${\hat{\Gamma }}_{n,1}=\underset{\tau }{\arg\max}R_{g1n}\left(\tau \right)$$

### 3.5. Refined Estimation Based on D-GDOP

Due to the spatial filtering and directional signal enhancement provided by beam forming, Equation (19) yields a high-precision time-difference estimate. Using this high-precision estimate, the following localization system of equations is constructed:

(31) $${\mathrm{Gp}}_{k}=h$$

where

(32) $$G=\left[\begin{array}{ccc} x_{2,1} & y_{2,1} & R_{2,1}\\ x_{3,1} & y_{3,1} & R_{3,1}\\ \vdots & \vdots & \vdots \\ x_{N,1} & y_{N,1} & R_{N,1} \end{array}\right],p_{k}=\left[\begin{array}{c} v_{k}\\ w_{k}\\ R_{1} \end{array}\right],h=\frac{1}{2}\left[\begin{array}{c} K_{2}-K_{1}-R_{2,1}^{2}\\ K_{3}-K_{1}-R_{3,1}^{2}\\ \vdots \\ K_{N}-K_{1}-R_{N,1}^{2} \end{array}\right]$$

where

(33) $$x_{n,1}=x_{n}-x_{1},y_{n,1}=y_{n}-y_{1},n=2,\ldots ,N$$

(34) $$R_{n,1}=R_{i}-R_{1}=\sqrt{{\left(v_{k}-x_{n}\right)}^{2}+{\left(w_{k}-y_{n}\right)}^{2}}-\sqrt{{\left(v_{k}-x_{n}\right)}^{2}+{\left(w_{k}-y_{n}\right)}^{2}}=c{\hat{\Gamma }}_{n,1}$$

(35) $$K_{n}=x_{n}^{2}+y_{n}^{2},n=1,\ldots ,N$$

The position estimate is obtained using the least-squares method as follows:

(36) $$p_{k}={\left(G^{T}\mathrm{WG}\right)}^{-1}G^{T}\mathrm{Wh}$$

where $W=diag(w_{2},w_{3},\cdots ,w_{N})$. Ideally, the weighting matrix W in WLS is determined by the covariance matrix of the measurement errors. However, in practice, this covariance is difficult to obtain as prior knowledge. Here, we compute the GDOP using the coarse estimate $p_{ini}$ and the array positions, and assign weights based on the D-GDOP, that is the varying contributions of different nodes to the overall GDOP.

**Figure 3:**
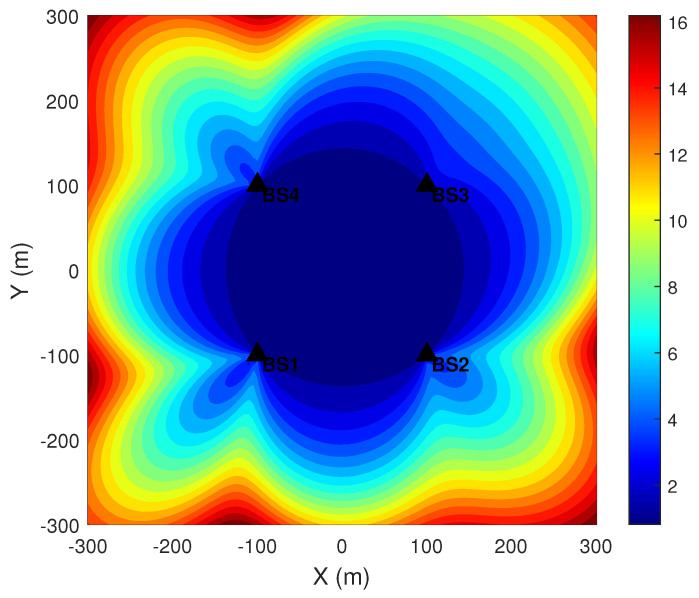
GDOP distribution for four-station TDOA localization.

GDOP quantifies the theoretical localization error as the source occupies different positions in space and serves as a key metric for evaluating the quality of the geometric configuration in localization. Figure 3 shows the GDOP distribution for a four-station TDOA localization scenario. It can be observed that certain areas in the figure exhibit large GDOP values, corresponding to unfavorable geometric configurations in the simulation. If the true position of the radiation source lies in an area with a high GDOP, the coordinate estimates will inevitably be affected.

The D-GDOP-based weighting scheme is designed to mitigate the adverse impact of geometrically unfavorable observation stations on localization accuracy. The specific weight assignment is as follows:

(37) $$w_{n}=GDOP_{-n}-GDOP_{all}$$

where ${GDOP}_{-n}$ denotes the GDOP calculated using all observation points except the *n*-th one, and ${GDOP}_{all}$ denotes the GDOP calculated using all observation points. The calculation of GDOP is given in [23].

The refined estimate can then be used again to compute updated D-GDOP-based weights, yielding an even more accurate position estimate.

The next row is then extracted from the matched angle matrix and the beam forming procedure is repeated to compute the coordinate estimate for the next source, continuing until coordinate estimates for all sources are obtained.

In summary, the proposed method is presented in Algorithm 1.

**Algorithm 1.** Proposed method**Input:** 
$X_{n}$
**Initialize:** l=1;   **Step 1** Use DCS-SOMP to obtain DOA estimates ${\hat{\alpha }}_{k,n}$;   **Step 2** Obtain matched angles ${\hat{\theta }}_{l,n}$ based on geometric consistency,       compute coarse estimates: $p_{ini,l}={\left({G_{ini,l}}^{T}G_{ini,l}\right)}^{-1}{G_{ini,l}}^{T}h_{ini,l}$;   **Step 3** Obtain the spectrum of the beam-formed received signal:       ${\hat{X}}_{l,n}\left(\omega \right)=w^{H}\left(\omega ,\theta_{l,n}\right)X_{n}\left(\omega \right)$;   **Step 4** Estimate the TDOA: ${\hat{\Gamma }}_{n,1}=\underset{\tau }{\arg\max}R_{g1n}\left(\tau \right)$   **Step 5** Compute the D-GDOP-based weight matrix W,       and obtain the refined estimate:$p_{l,re}={\left(G^{T}\mathrm{WG}\right)}^{-1}G^{T}\mathrm{Wh}$   **Step 6** Return to Step 5, recompute the D-GDOP-based weights $W_{\mathrm{re}}$ using $p_{l,re}$,       and obtain the final position estimate:$p_{l}={\left(G^{T}W_{\mathrm{re}}G\right)}^{-1}G^{T}W_{\mathrm{re}}h$;   **Step 7** If l≤K, then l=l+1, and go to Step 2. Otherwise go to Output;**Output:** $p_{k}\left(k=1,2,\cdots ,K\right)$.

## 4. Simulation Result

This section employs Monte Carlo simulation experiments to evaluate the performance of the algorithm in estimating the positions of radiation sources.

**Figure 4:**
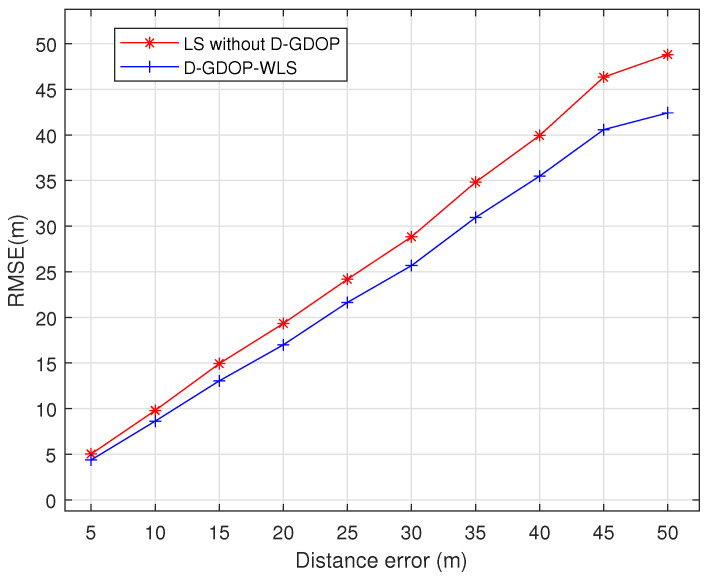
Comparison of performance of the D-GDOP-WLS against the LS without D-GDOP.

We first verify the effectiveness of the proposed D-GDOP weighting scheme. Figure 4 compares the performance of the D-GDOP-WLS method against the conventional LS method under different ranging errors—defined as the time-difference estimation error multiplied by the signal propagation speed. The simulation parameters are set as follows: the radiation source is located at (3000,6000) m, respectively; the number of observation stations is N=5, positioned at $u_{1}=\left[0,0\right]$ km, $u_{2}=\left[0,4\right]$ km, $u_{3}=\left[5,0\right]$ km, $u_{4}=\left[7,7\right]$ km, and $u_{5}=\left[4,8\right]$ km. It is clearly observed that the conventional LS method, which does not account for geometric configuration effects, yields larger errors. In contrast, the D-GDOP-WLS method achieves superior performance by mitigating the adverse impact of geometrically unfavorable observation stations on localization accuracy.

**Figure 5:**
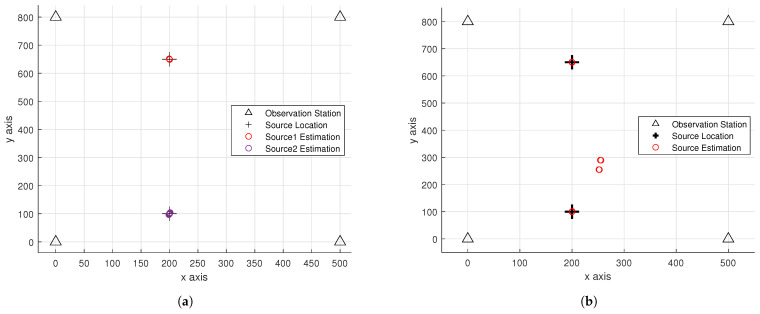
(**a**) Scatter plot of the proposed algorithm. (**b**) Scatter plot of the LS method without source separation.

Figure 5 compares the scatter plots of the proposed algorithm with TDOA localization method that does not use source separation. The left subplot shows the localization scatter plot of the proposed method, while the right subplot displays the result obtained by the LS method without source separation. The simulation parameters are set to SNR=−10 dB and Fs=8000 MHz. It can be seen that the method without source separation has a very low accuracy rate, whereas the proposed method can correctly separate the targets and achieve precise localization. This demonstrates the effectiveness of the source separation in the proposed algorithm.

**Figure 6:**
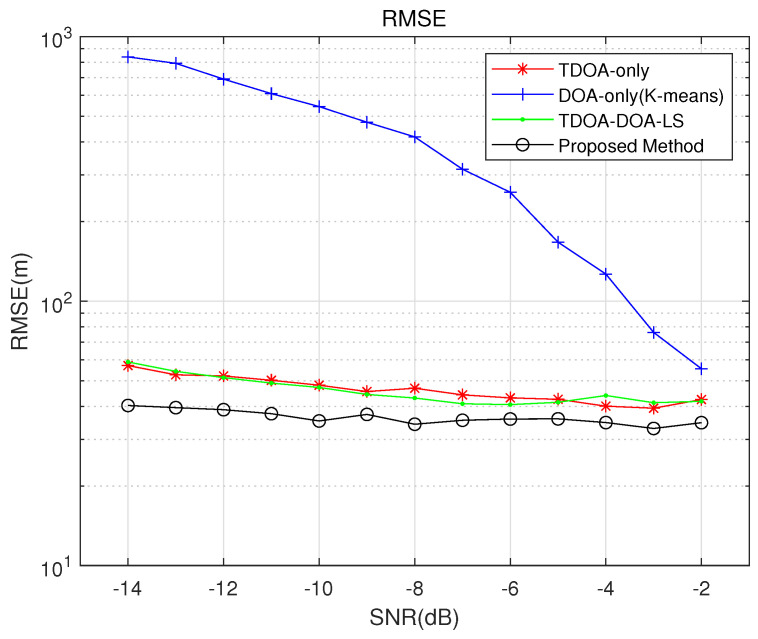
Comparison of localization performance of different algorithms versus SNR.

Figure 6 investigates the localization performance of the proposed algorithm compared with several other methods under varying SNR. The simulation parameters are set as follows: the two radiation sources are located at (1000, 600) m and (1100,1400) m, respectively; the number of observation stations is N=4, positioned at $u_{1}=\left[0,0\right]$ m, $u_{2}=\left[0,100\right]$ m, $u_{3}=\left[100,0\right]$ m, and $u_{4}=\left[100,100\right]$ m; each station is equipped with a uniform linear array consisting of M=4 elements; and the sampling frequency is $f_{s}=8000$ MHz. The number of Monte Carlo trials is 500. The benchmark algorithms include the K-means clustering algorithm, the CHAN algorithm after multi-target data matching, and the TDOA-DOA joint least-squares method after multi-target data matching.

Simulation results show that the proposed algorithm achieves a lower RMSE than the other methods. The joint least-squares method offers localization accuracy comparable to that of the CHAN algorithm; however, due to inherent limitations in its formulation, it cannot improve accuracy by incorporating redundant measurements and is thus constrained by the fundamental precision limits of TDOA-based localization. The conventional K-means clustering algorithm outperforms the proposed method at high SNR, but its performance degrades significantly as the SNR decreases. In contrast, the proposed algorithm effectively mitigates performance degradation in low-SNR conditions. Moreover, by employing beam forming to suppress interference from other directions, the proposed method resolves the TDOA association ambiguity in multi-source scenarios, enhances the output SNR of the array, and thereby achieves more accurate time-delay estimates. Consequently, The localization accuracy has improved by approximately 20% compared to both the CHAN algorithm and the joint least-squares method.

**Figure 7:**
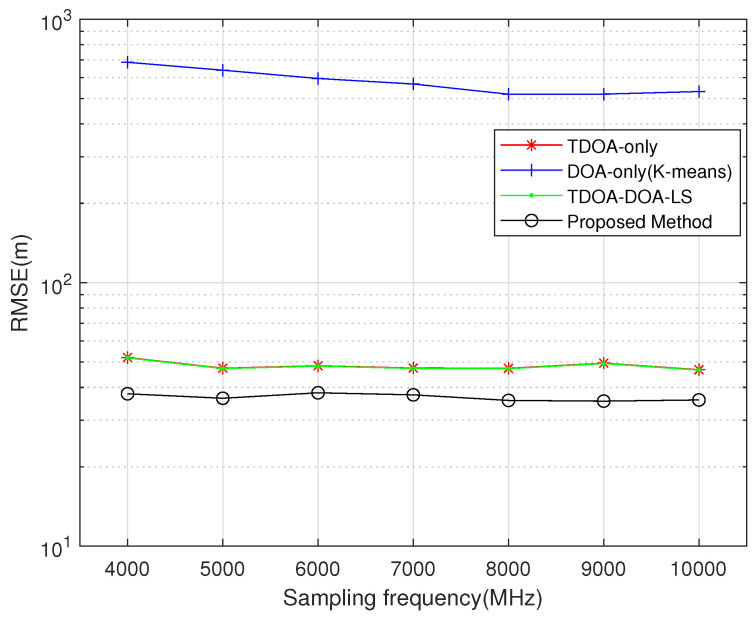
Comparison of localization performance of different algorithms versus sampling frequency.

Figure 7 investigates the localization performance of the proposed algorithm compared with several other algorithms under different sampling rates, with the SNR fixed at –10 dB and all other parameters kept unchanged. As shown in the figure, the proposed algorithm achieves lower localization error than the other methods. Moreover, compared to the competing algorithms, The proposed algorithm exhibits the smallest increase in RMSE, amounting to only 38% of the increase observed with the TDOA-DOA joint algorithm, indicating its superior robustness.

## 5. Conclusions

By leveraging both DOA and TDOA measurements, this paper proposes a cascaded DOA-TDOA-based multi-source weighted localization method. The approach first obtains a coarse estimate using DOA, then performs wideband spatial filtering via beam forming to separate multiple sources before carrying out TDOA estimation. Pseudo-linear equations are subsequently formulated, and a WLS estimator is applied with weights determined by D-GDOP to obtain a refined estimate. Finally, this refined estimate can be used to iteratively update the weights, yielding an even more accurate localization result.

Compared with other multi-source TDOA localization methods, the proposed approach effectively combines the strength of DOA-based localization in separating sources in multi-source scenarios with the high accuracy of TDOA-based localization in single-source cases—without requiring explicit TDOA data association. Moreover, it fully exploits the array’s beam pattern characteristics, leading to improved localization accuracy. The simulation results demonstrate the effectiveness of the proposed method. 

## Data Availability

Data are contained within the article.
